# Nutrient combinations exhibit universal antianxiety, antioxidant, neuro-protecting, and memory-improving activities

**DOI:** 10.3389/fnut.2022.996692

**Published:** 2023-01-06

**Authors:** Bangcheng Zhao, Xiuzhen Jia, Haotian Feng, Cheng Tang, Yixin Huang, Zifu Zhao, Jingyu Hao, Haoqiu Li, Jinrui Du, Yan Liu, Xingyu Bao, Zhihui Zhong, Yingqian Zhang, Qixing Zhong

**Affiliations:** ^1^Laboratory of Non-Human Primate Disease Modeling Research, State Key Laboratory of Biotherapy, West China Hospital, Sichuan University, Chengdu, China; ^2^Inner Mongolia Dairy Technology Research Institute Co., Ltd., Yili Innovation Center, Inner Mongolia Yili Industrial Group Co., Ltd., Hohhot, China; ^3^Sichuan SAFE Pharmaceutical Technology Co., Ltd., Chengdu, China; ^4^Department of Biological Sciences, College of Biological Science and Technology, Agricultural University of Hunan, Changsha, China; ^5^Sichuan Kangcheng Biotech Co., Inc., Chengdu, China

**Keywords:** nutrients, zebrafish, antianxiety, antioxidant, neuroprotection, memory-improving

## Abstract

Anxiety disorders are the most common mental disorders and, without proper treatment, may lead to severe conditions: e.g., somatic disorders or permanent damage to central nervous system. Although there are drugs in clinical trials, this study focuses on exploring the efficacy of nutrients in treating these diseases. We built different zebrafish models and screened several nutrient combinations for their antianxiety, antioxidant, neuro-protecting, and memory-improving activities. Our results showed that the combinations of nutrients (e.g., Walnut Peptides + Theanine at 14.2 + 33.3 μg/ml) have similar or better activities than the positive control drugs. In addition, we discovered that the effects of the nutrients in the above four aspects were universal and highly related. This study is noteworthy as it suggested that nutrients could be healthier and greener drug alternatives and provide similar or better universal treatments for anxiety and related conditions.

## 1. Introduction

Anxiety disorders are the most common mental disorders that generally begin before or during early adulthood. Major characteristics of anxiety disorder include excessive fear and anxiety about threats or their prevention. In this condition, the brain circuits that respond to danger are dysfunctional. Anxiety disorders are influenced by genetic, environmental factors and epigenetic interactions. It is also common for anxiety disorders to be comorbid with other somatic disorders and mental disorders (e.g., depression) (1). Unlike the state of anxiety, anxiety disorders impair individuals’ daily life performance, resulting in high costs of public health care worldwide (2). According to the ICD-10 classification, there are eight types of anxiety disorders. Among them, specific (isolated) phobias are the most common anxiety disorders, with a 12-month prevalence of 10.3%, followed by social anxiety disorder (SAD, also called social phobia; 2.7%) and general anxiety disorder (GAD; 2.2%) (3, 4). While there continues to be extensive research on treating other mental disorders (e.g., depression and schizophrenia) (5), new treatments for anxiety disorders are still needed.

In addition, anxiety disorders are known to be associated with brain damage (6). Effective brain-protective strategies are thus needed during the disease’s progression. Up to now, no specific drug is available to remission the symptoms of these diseases. Nevertheless, nutritional samples supplemented with food extracts were reported to have such effects recently (7). In addition, studies have shown that nutrients can often outperform drugs in achieving health goals as they do without any side effects, with which prescription drugs are often riddled (8). Recently, the use of nutrients such as tryptophan, vitamin B6, B12, folic acid, and glutamic acid has drawn much attention in the research of anti-depression and antianxiety (7, 9). Despite this, identifying more nutrients for their antianxiety and neuroprotective activities is still needed and requires low-cost and high-throughput screening techniques (9).

In this study, we built anxiety, oxidative stress (OS), nervous system injury (NSI), and Alzheimer’s disease (AD) zebrafish models. With these models, we investigated several nutrients and combinations for their antianxiety, antioxidant, neuro-protecting and memory-improving capabilities. Our results demonstrated that using nutrient combinations (e.g., Walnut Peptides + Theanine at 14.2 + 33.3 μg/ml) as healthier and greener drug alternatives could provide similar or better universal treatments for anxiety and related conditions.

## 2. Materials and methods

### 2.1. Experimental model animals

In this study, wild-type AB strain zebrafish were bred in natural pairs. Zebrafish aged four days post-fertilization (dpf) were chosen for antianxiety activities and memory-improving [responsiveness and acetylcholinesterase (AChE) production] evaluation. Zebrafish aged one dpf were used to evaluate the samples’ protective abilities to the central nervous system (CNS). Translucence melanin allele of Albino-type zebrafish bred in natural pairs aged three dpf were used to evaluate the antioxidant efficacy.

All zebrafish were raised in 28°C fish farming water (water quality: 200 mg of instant sea salt per 1 L reverse osmosis water, conductivity: 450–550 μS/cm; pH: 6.5–8.5; hardness: 50–100 mg/L CaCO_3_) as culture, license number of experimental animals used: SYXK (Zhejiang) 2022-0004. The feeding practice was carried out following the requirements of the international AAALAC certification (certification number: 001458).

### 2.2. Model establishment and grouping

The anxiety zebrafish model was established by adding 1-(3-chlorophenyl) piperazine hydrochloride (mCPP, 20.0 μg/mL, 1 h incubation at 28°C and washed off) (10, 11). After that, in the positive drug group, Selegiline was added (20.0 μg/ml, 24 h incubation at 28°C). The OS model was established by adding menadione (2.25 μM, 22 h incubation at 28°C) to the culture. In the positive drug group, N-Acetyl-L-cysteine (NAC) was also added (62.5 μg/ml, 22 h incubation at 28°C). The zebrafish NSI model was established by adding Mycophenolate mofetil (0.4 μM, 48 h incubation at 28°C) to the culture. In the positive drug group, Glutathione was also added (615 μg/ml, 48 h incubation at 28°C). The zebrafish AD model was established by adding aluminum chloride (140 μM, 24 h incubation at 28°C) to the culture. Donepezil hydrochloride was also added to the positive drug group (3.33 μg/ml, 24 h incubation at 28°C). The nutrients in each test were incubated under the same condition as their respective positive drug group. The control (blank control, culture-only) was set up simultaneously with the other groups.

### 2.3. Sample information

Four positive drugs were used in this experiment: Selegiline, NAC, Glutathione, and Donepezil hydrochloride are commercially available synthetic chemicals. The nutrients, including Stachyose, Lactic acid bacteria-*Lactobacillus Plantarum* (PS128), Walnut Peptide (WP), Desert Cistanche (DC), and Theanine (Th) were all plant extracts. Casein Peptides (CP) were originated from milk proteins. All the nutrients are commercially available. Their combinations were nutrient samples with determined concentrations. The names and concentrations of the positive drugs, individual nutrients and combinations tested were summarized in Table 1. Their doses in zebrafish and the estimated doses in humans were also listed.

**TABLE 1:** The names, origins and doses of the samples tested.

Group	Name	Origin	Human dose (estimated)	Zebrafish dose (μg/ml)
**Positive drugs**	Selegiline	Chemical substance (Orion Corporation, Finland)	N.A.	20
	N-acetyl-L-cysteine (NAC)	Chemical substance (Shanghai Aladdin Biochemical Technology Co., Ltd)	N.A.	62.5
	Glutathione	Chemical substance (Sigma)	N.A.	615
	Donepezil hydrochloride	Chemical substance (Eisai China Inc.)	N.A.	3.33
**Nutrients**	Stachyose	The plant extract, a galactose derivative of sucrose (Xi’an APP-Chem (Tech) Co., Ltd)	3 g/day	500
	Lactic acid bacteria-*Lactobacillus Plantarum* (PS 128)	Traditional fermented mustard products of Taiwan (Asian Probiotics and Prebiotics corporation)	1.8 × 10^12^CFU/g	1,000
	Walnut peptides (WP)	The plant extract, bioactive peptide extracted from the protein of walnut residues (Sinphar group Co.)	0.75 g/day	125
	Desert cistanche (DC)	The plant extract, tonic traditional Chinese medicine (Sinphar group Co.)	0.75 g/day	125
	Casein peptides (CP)	Milk (Guangdong Huapeptides Biotechnology Co., Ltd.)	0.6 g/day	100
	Theanine (Th)	The natural amino acid in green tea (Shanghai Novanat Co., Ltd)	400 mg/day	66.6
**Combinations**	Stachyose + PS128	N.A.	0.5 g/day + 9 × 10^11^ CFU/g	83.3 + 500
			1.5 g/day + 9 × 10^11^ CFU/g	250 + 500
			3 g/day + 9 × 10^11^ CFU/g	500 + 500
	WP + DC	N.A.	0.375 + 0.375 g/day	62.5 + 62.5
			0.1875 + 0.562 g/day	31.2 + 93.7
			0.562 + 0.1875 g/day	93.7 + 31.2
	WP + CP	N.A.	0.085 + 0.15 g/day	14.2 + 25
			0.17 + 0.3 g/day	28.4 + 50
			0.34 + 0.6 g/day	56.7 + 100
	WP + Th	N.A.	85 + 200 mg/day	14.2 + 33.3
			170 + 400 mg/day	28.4 + 66.6

N.A, not applicable, CFU, colony-forming unit.

### 2.4. Zebrafish behavioral test

In this test, four dpf wild-type AB zebrafish were randomly selected and raised in the six-well plates with 30 fish per well (for all groups). After treatments, all zebrafish were transferred into a 96-well plate (one tail/well) and 200 μl of culture solution was added to each well. A behavioral analyzer was then used to detect the total moving distance of zebrafish for further analysis.

### 2.5. Cortisol production measurement

In this test, the wild-type AB strain zebrafish of four dpf were randomly selected and raised in the six-well plate, with 30 zebrafish per well (for all groups). After treatments, the cortisol concentration of zebrafish (three replicates per treatment) was measured using the enzyme-linked immunosorbent assay (ELISA kit, Supplementary Table 1: Instruments and consumables). The data were then collected and analyzed.

### 2.6. Antioxidant effect evaluation

The translucent albino zebrafish with three dpf melanin allele mutations were randomly selected and raised in six-well plates. Thirty zebrafish were kept in each well (for all groups). After treatments, zebrafish were transferred into 24-well plates and stained with specific reactive oxygen species (ROS) fluorescent dye. After staining, 10 zebrafish were randomly selected from each test group and placed under a fluorescence microscope for imaging. Nis-elements D 3.20 advanced image processing software was used for analysis. The fluorescence intensity at the zebrafish yolk sac was measured for further analysis.

### 2.7. CNS protective effect evaluation

Zebrafish of the wild-type AB strain of one dpf were randomly selected and raised in six-well plates with 30 zebrafish per well (for all groups). After treatments, zebrafish were stained with acridine orange (AO, Supplementary Table 1: Instruments and consumables). After staining, 10 zebrafish were randomly selected from each group and placed under a fluorescence microscope for imaging. Images were processed by Nis-elements D 3.20 advanced image processing software. After that, the fluorescence intensity in the zebrafish CNS was measured.

### 2.8. Memory improvement

#### 2.8.1. Responsiveness

The wild-type AB strain of four dpf zebrafish was randomly selected and raised in 6-well plates, 30 zebrafish per well (for all groups). After treatments, 10 zebrafish in each group were randomly selected and studied. The behavioral analyzer measured the zebrafish’s average moving speed per minute under light and in dark environments for further analysis.

#### 2.8.2. AChE measurement

Zebrafish of the wild-type AB strain of four dpf were randomly selected and raised in six-well plates with 30 zebrafish per well (for all groups). After treatments, an AChE assay kit was used to analyze zebrafish by ELISA. The AChE-fluorescence value was measured for further analysis.

### 2.9. Statistical analysis

Statistical analysis was conducted using SPSS v.20 software (IBM Ltd., UK). Data following normal distributions were represented as means ± standard errors of the mean (SEM). Differences among groups were analyzed by one-way ANOVA, followed by the *post hoc* Tukey Dunnett’s multiple comparison test. In the non-parametric test, the Kruskal–Wallis single-factor ANOVA test (K samples) was used. Comparative analysis of the two groups: independent *t*-tests and non-parametric tests were used for normal distributions. Mann–Whitney tests were used for non-normal distributions. The correlations between each effect were analyzed statistically by Pearson’s correlation. The area under curve (AUC) of velocity in 20 min was used for correlations between reactivity and other effects. When putting all the indexes together in one graph, we normalized every index to control to compare the full impact of each combination. *P* values less than 0.05 were considered significant in all analyses. Data were collected from at least three biological replicates.

## 3. Results

### 3.1. The nutrient combinations exhibited antianxiety effects

**FIGURE 1:**
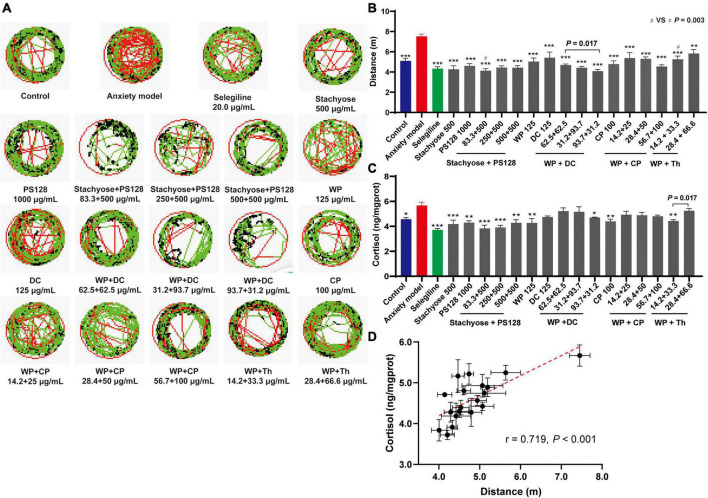
Antianxiety effects of nutrient combinations. **(A)** Representative trace map of the zebrafish behavioral tests (*n* = 10). The moving speed <4, 4–20, and >20 mm/s were marked in green, black, and red lines, respectively. **(B)** Moving distance of zebrafish in each group (*n* = 10). **(C)** Cortisol production level of zebrafish in each group (zebrafish in three wells were combined and measured as one sample, *n* = 3). **(D)** The cortisol production level of zebrafish in each group was plotted against the distance measured in the behavioral test. n = 3. Data in panels **(B–D)** were presented as mean ± SEM. *P < 0.05, **P < 0.01, ***P < 0.001 compared with anxiety model. In panel **(B)**, Stachyose + PS128 (83.3 + 500 μg/ml) **(#)** vs. WP + Th (14.2 + 33.3 μg/ml) (#), *P* = 0.003. Data were collected from three biological replicates.

We used mCPP to increase zebrafish’s anxiety-like behaviors and stimulate the production of cortisol (10, 12). The zebrafish’s movement (representative trace maps in Figure 1A and Supplementary Figure 1A) reflected the zebrafish’s anxiety level (13). From the results in Figure 1B and Supplementary 1B, the distance in the model group was significantly longer than the control (*P* < 0.001), indicating that the zebrafish anxiety model was effectively established. As expected, zebrafish exhibited a shorter distance in the Selegiline (an irreversible monoamine oxidase (MAO)-B inhibitor that increases 5-HT levels) (14) group than in the model or the control. Based on the preliminary screening (Supplementary Figure 1B), we selected several nutrients and their combinations for further tests.

Figure 1B revealed that all the individual nutrients and combinations exhibited shorter distances than the model group, indicating their antianxiety effects. Among them, Stachyose at 500 μg/mL was the most effective nutrient, with comparable activity to the positive drug. For the combinations, firstly, we noticed that when the ratio of WP + DC was adjusted based on weight (1:1, 1:3, and 3:1), their effects varied and, at 93.7 + 31.2 μg/ml (this concentration was selected for downstream tests) showed the highest capability which is comparable to the positive drug. Secondly, the effects of the combinations were dose-specific but not dose-dependent in all groups. For instance, in Stachyose + PS128 group and WP + Th group, the increase of both components’ concentrations did not promote their antianxiety capabilities. Thus, in Stachyose + PS128 and WP + Th groups we selected the lowest concentrations (83.3 + 500 and 14.2 + 33.3 μg/ml, Stachyose + PS128 is more effective, *P* = 0.003, Figure 1B) for downstream tests. Nevertheless, the concentration increases in the combination of WP + CP promoted the antianxiety effect, showing a preliminary dose dependence.

The production of cortisol in each group was also measured to show the zebrafish’s anxiety level (15). As expected, most tested nutrients and combinations showed lower cortisol levels than the model group, with Stachyose + PS128 (83.3 + 500 μg/ml) being the most effective combination (Figure 1C). Surprisingly, specific concentrations of WP + DC and WP + CP combinations did not inhibit the production of cortisol, which was not consistent with the zebrafish’s moving distance. Despite this, a positive correlation (*r* = 0.719, *P* < 0.001) was still observed between the distance and the cortisol level (Figure 1D), showing that the antianxiety effects assessments were reliable.

### 3.2. The nutrient combinations were effective in antioxidation

**FIGURE 2:**
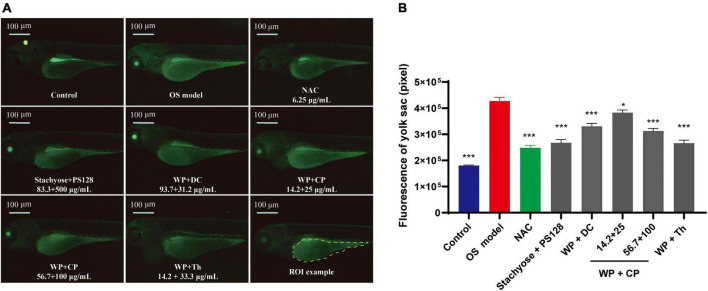
Antioxidant effects of nutrient combinations. **(A)** The representative images of yolk sac fluorescence in each group. **(B)** The fluorescence intensity of the yolk sac in each group (*n* = 10). Data were presented as mean ± SEM. *P < 0.05, ***P < 0.001 compared with OS model. Data were collected from three biological replicates.

It was reported that biological processes such as oxidative stress are partly affected by mood states (16). Therefore, we continued to investigate the antioxidant effects of the nutrients. Menadione was used to induce oxidative stress, which triggered green fluorescence expression in the yolk sac of zebrafish (Figure 2A). The increase in fluorescence intensity represents a decrease in antioxidant function (17). The intensity (Figure 2B) was observed to be much higher in the model than in the control, indicating that the model establishment was successful. All the combinations showed antioxidant effects. Although none of them could recover the model to the level of the control, Stachyose + PS128 and WP + Th combinations showed similar effects as the positive control drug NAC, a glutathione precursor, which modulates glutamatergic transmission and targets oxidative pathways (18). Their antioxidant effects were also affected by concentrations: in WP + CP, the higher concentration at 56.7 + 100 μg/ml was more effective than the lower concentration, indicating a preliminary dose-dependence (Figure 2B).

### 3.3. The nutrient combinations showed central neuroprotective effects

**FIGURE 3:**
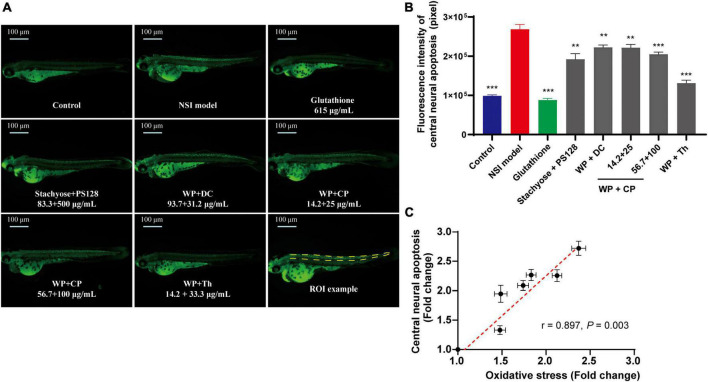
Central neuroprotective effect of nutrient combinations. **(A)** The representative fluorescence images of central neural apoptosis in zebrafish. **(B)** The quantification of fluorescence intensity of central neural apoptosis in each group. *n* = 10, **P < 0.01, ***P < 0.001. **(C)** The oxidative stress (from Figure 2B, value was normalized to the control) was plotted against the central neural apoptosis level (normalized to the control), (*r* = 0.897, *P* = 0.003). Data in panels **(B,C)** were presented as mean ± SEM. **P < 0.01, ***P < 0.001 compared with NSI model. Data were collected from three biological replicates.

It was also reported that there are links between anxiety, oxidative stress and neuron damage (19, 20). Thus, we continued to investigate the central neuroprotective effect of nutrients. Mycophenolate mofetil was used to induce NSI in zebrafish, which triggered green fluorescence (Figure 3A) in apoptotic cells at CNS (21). Firstly, in the model group, the fluorescence intensity (Figure 3B) was almost three times higher than the control, showing the model establishment was successful. Secondly, the combination of WP + Th showed the strongest activity in reducing central neural apoptosis, close to the positive drug Glutathione, whose depletion is a common feature of apoptotic cell death (22). Unlike the antianxiety tests, the other combinations (WP + DC, WP + CP, and Stachyose + PS128) exhibited a similar but much lower protective effect than WP + Th. A preliminary dose-dependent effect was observed in WP + CP and further tests with more concentrations are needed. Noteworthily, when we plotted the antioxidant effect of the nutrients against their central neural apoptosis level, a good positive correlation was established (*r* = 0.897, *P* = 0.003, Figure 3C), showing that both effects from the nutrients were highly related.

### 3.4. The nutrient combinations improved zebrafish’s memory

**FIGURE 4:**
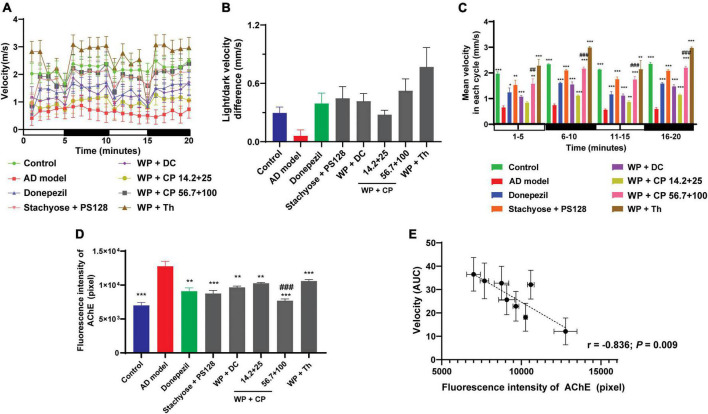
Memory-improving effects of nutrients. **(A)** The lines represented the average moving speed of zebrafish per minute under light and in dark environments for 20 min (*n* = 12). **(B)** The difference in velocity in light (0–5 and 10–15 min) and dark (5–10 and 15–20 min) was measured (*n* = 12). **(C)** The histogram of zebrafish’s average movement velocity in each cycle (5 min per cycle, *n* = 12). **(D)** The fluorescence intensity of AChE in each group (*n* = 6). **(E)** The intensity of AChE was plotted against the AUC of the mean velocity from panel **(A)** (*n* = 6, *r* = –0.836, *P* = 0.009). Data in panels **(B,C)** were presented as mean ± SEM. *P < 0.05, **P < 0.01, ***P < 0.001 compared with AD model; ^##^*P* < 0.001, ^###^*P* < 0.001 compared with WP + CP (14.2 + 25 μg/ml). Data were collected from three biological replicates.

Apart from the above tests, we further investigated the memory-improving abilities of the nutrients, as anxiety was reported to cause memory damage (23). During model establishment, aluminum chloride was used to build zebrafish’s AD model: Excessive intake of aluminum chloride causes toxic effects on the nervous system, resulting in abnormal zebrafish behavior (24). It also destroys the cholinergic nerve function, increases the production of amyloid, reduces learning ability and causes memory loss (24, 25). Donepezil hydrochloride, an AChE inhibitor, was used as the positive drug in this test. The nutrients’ memory-improving abilities were evaluated by measuring the velocity of zebrafish using the response capability test (26). From the graph shown in Figure 4A, the mean velocity of the model group was the lowest of all, demonstrating the effectiveness of the modeling. The results showed that the nutrients improved the mean velocity, which was slower and less stable under light (0–5 and 10–15 min) than in the dark (5–10 and 15–20 min). WP + CP significantly improved zebrafish’s response capability, with a preliminary concentration-dependent effect. It is noteworthy that WP + Th showed the most substantial capability in improving velocity, even recovered to a higher level than the control.

Furthermore, zebrafish with nervous system damage were reported to have slower swimming speed; But after treatment, their swimming speed was recovered with the stimulation of light-dark alternation (27). Consistent with this report, the difference in velocity between light and dark was much higher in nutrient combinations (e.g., WP + Th) than in the model (Figure 4B). The mean velocity was further broken down into cycles of 5 min in Figure 4C. The patterns of the results in each period were similar, further proved that the effects of nutrients in the shorter period are consistent. Another crucial memory-improving index was the production of AChE (28), the intensity of which (Figure 4D) confirmed the effectiveness of all the combinations, with WP + CP at a higher concentration being the most effective. When we plotted the AChE intensity against the AUC of the mean velocity of all groups, a good negative correlation was observed (*r* = −0.836, *P* = 0.009, Figure 4E), indicating the memory-improving assessments were reliable.

### 3.5. The effects of nutrient combinations were universal and highly-related

**FIGURE 5:**
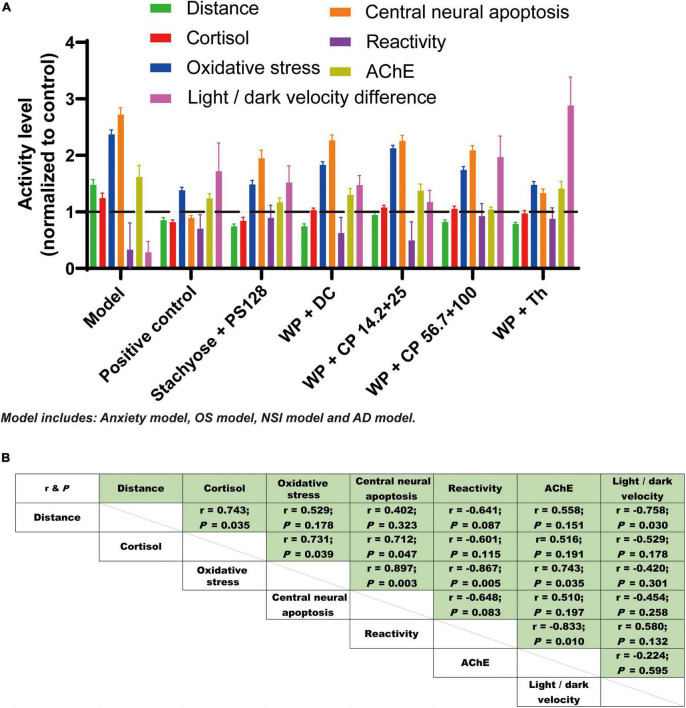
The effects of nutrient combinations were universal and related. **(A)** Results of all the effects from the test groups were consolidated and presented in bar chat. The four models, including anxiety, OS, NSI and AD, were shown as “Model” for short. Data were normalized to the control (value of which was normalized to 1) of each test and presented as mean ± SEM. **(B)** The effects tested from test groups were plotted against each other. The r-value and *P*-value of each plot are shown in panel **(B)**.

With all these data in hand, we further analyzed the relationships between different effects of the nutrient combinations. The results of all effects were consolidated in Figure 5A, from which we observed that the pattern of the effects from test groups are similar and most of the nutrients improved their respective models. Among them, WP + Th was almost the most promising in all tests. When the value of individual effect was plotted against each other, good correlations (17 out of 21 have absolute *r* value larger than 0.5, representative plots were shown in Supplementary Figure 2) were observed (Figure 5B), suggesting the effects of the nutrient combinations were universal and highly related.

## 4. Discussion

In this study, we used different zebrafish models to identify nutrient combinations as potential drug alternatives for treating anxiety and related conditions. In the antianxiety test, Stachyose + PS128 (83.3 + 500 μg/ml) exhibited similar capability (Figures 1B, C) as the positive drug Selegiline. Stachyose is a soluble carbohydrate that works as a probiotic that reduces the negative impacts on antibiotic-destroyed microbiota (29) and improves long-term potentiation impairment caused by stress through the gut-brain axis (30). PS128 improved stress, anxiety and insomnia in humans (31). It could serve as a therapeutic adjuvant for treating major depressive disorder (32). These effects might be related to ameliorated inflammation and oxidative stress through microbiota modulation and related metabolites (33). In preliminary screening, PS128 at 500 μg/ml showed an antianxiety effect (Supplementary Figure 1), and Stachyose is reported to be an absorption enhancer (34). Therefore, we speculate that PS128 is the more effective component while Stachyose plays an auxiliary role.

In the downstream antioxidant, neuro-protecting and memory-improving tests, three positive drugs rescued their respective models via different mechanisms of action. Our study showed that WP + Th, with a relatively lower concentration of 14.2 + 33.3 μg/ml, was the most effective in the above three tests, with similar or better activities than these drugs. WP has been reported to promote sleep quality by regulating neurotransmitters in the brain tissue in mice (35). It could also enhance memory, cognition and improve sleep quality in clinical trials (36). Th, a non-protein amino acid abundantly present in tea leaves, has a similar structure as glutamate and glutamine that has been shown to improve anxiety, sleep and cognitive function, as well as reduce oxidative stress (37). The mechanism might be associated with its cerebro-protective effect, the neuroprotective effect and its glutamine carrier for inhibiting the combination of extracellular glutamine with neurons (38) or regulating the gamma-aminobutyric acid (GABA) receptor (39). We thus speculate that WP + Th might have a synergistic regulatory effect on neurotransmitters in the brain. Furthermore, in the aluminum chloride-induced AD model, the WP + Th group showed the best memory-improving abilities, even better than the control (Figures 5A–C), indicating WP + Th may have a potential effect on improving memory in other animals or even in the human context, which implies a great commercial value in the memory-improving food market.

In this study, we found a significantly high correlation between distance and light/dark velocity (Figure 5B), which was in line with the previous research (40). Oxidative stress is the essential pathophysiological process in hypoxia and aging. It causes glial activation, neurodegeneration, neurotransmitter/receptor dysregulation and induces CNS disorders (depression, anxiety, and dementia) (41). In our study, positive correlations were also found between oxidative stress and central neural apoptosis, as well as oxidative stress and AChE level, indicating that oxidative stress, central neural apoptosis and AChE production were reciprocal causation in the anxiety process. Noteworthily, not in many studies were these four effects studied together, let alone analyzing the correlations between them. In the following studies, we will further investigate the relationships between these four effects and the causation.

Zebrafish are widely used in CNS-related studies as they have human-like CNS: similar main structure of the brain, identical physiology and neurochemistry, etc. (42). Studies also compared the zebrafish and mouse models of anxiety. They also proved the predictive power of zebrafish for behavioral research (43). Since mammals are more genetically and physiologically similar to humans (44), further animal studies using rodents and large animals are also needed prior to human trials.

As most animal behavioral tests are subjective and unstable (45), using different behavioral tests to evaluate and recognize emotions is recommended. However, due to time constraints, only two behavioral tests were used in this study to assess anxiety and memory loss. We couldn’t perform more cognitive tests like the novel dark diving test, the T-maze test, or the object recognition test. In the following rodents experiment, we would select more behavioral tests to evaluate the nutrients and combinations on anxiety, recognition and memory.

## Data availability statement

The original contributions presented in this study are included in the article/Supplementary material, further inquiries can be directed to the corresponding authors.

## Author contributions

XJ, YZ, and QZ designed the experiments. BZ, CT, YH, JD, and XB performed the experiment. HF and ZiZ analyzed the data. ZhZ, JH, HL, and YL contributed to the reagents, nutrients, and nutrient combinations. BZ, YZ, and QZ prepared the manuscript. All authors have read and approved the final manuscript.
