# Supplementary material for: Nutrient combinations exhibit universal antianxiety, antioxidant, neuro-protecting, and memory-improving activities
Source: Front Nutr. 2023 Jan 6;9:996692. doi: 10.3389/fnut.2022.996692 (PMC9852889; doi:10.3389/fnut.2022.996692)
Supplement: Supplementary file 1 [file Data_Sheet_1.docx]

**Supplementary materials**


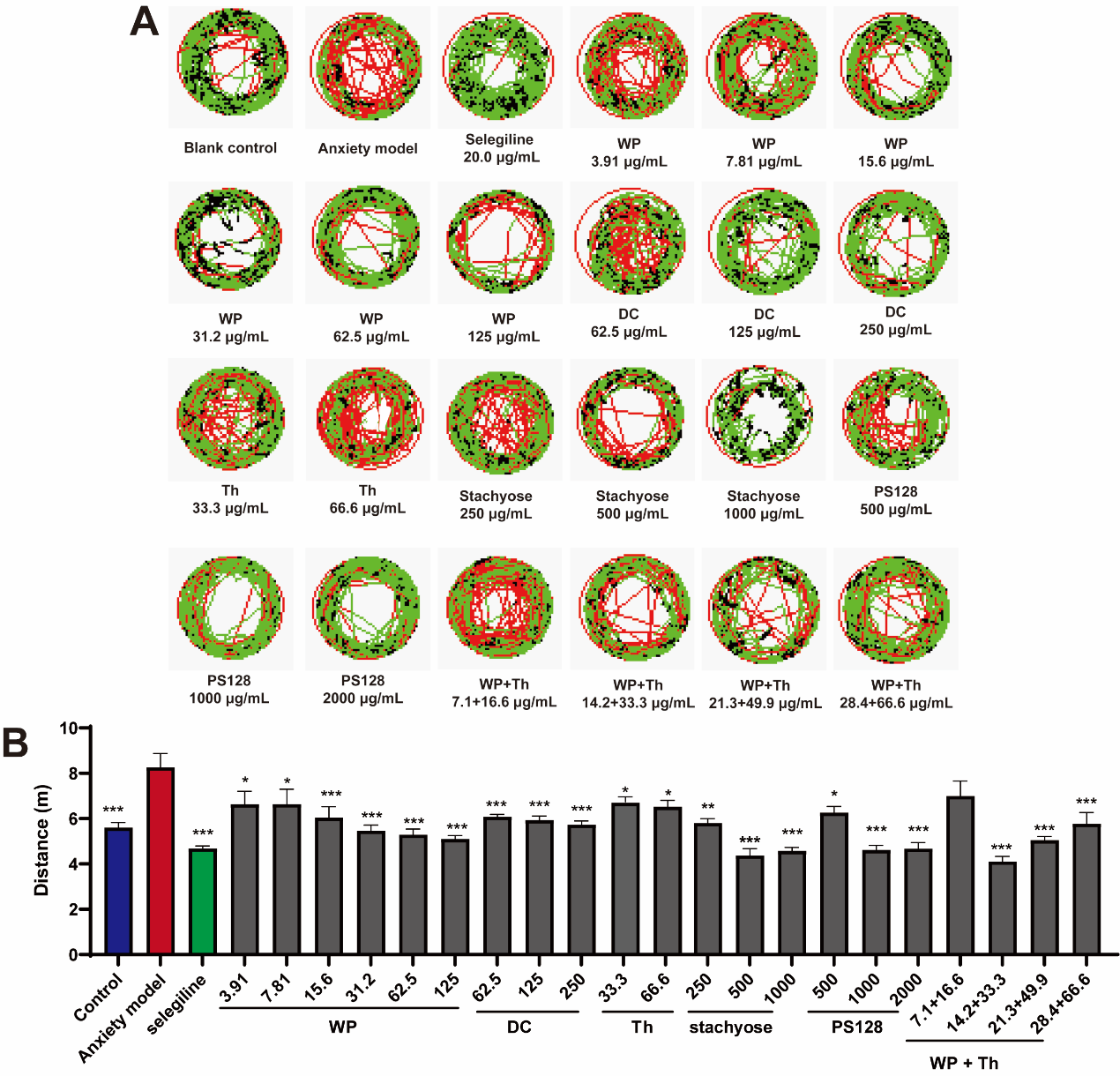


**Supplementary Fig. 1: Preliminary screening of nutrients with zebrafish behavioral test.** A. Trace map of first phase behavior test. The moving speed < 4 mm/s, 4-20 mm/s, and > 20 mm/s were marked in green, black and red lines, respectively. B. Moving distance of zebrafish in each group (n = 10). Data were presented as mean ± SEM. **P* < 0.05, ***P* < 0.01, ****P* < 0.001 compared with anxiety model. Data were collected from 3 biological replicates.


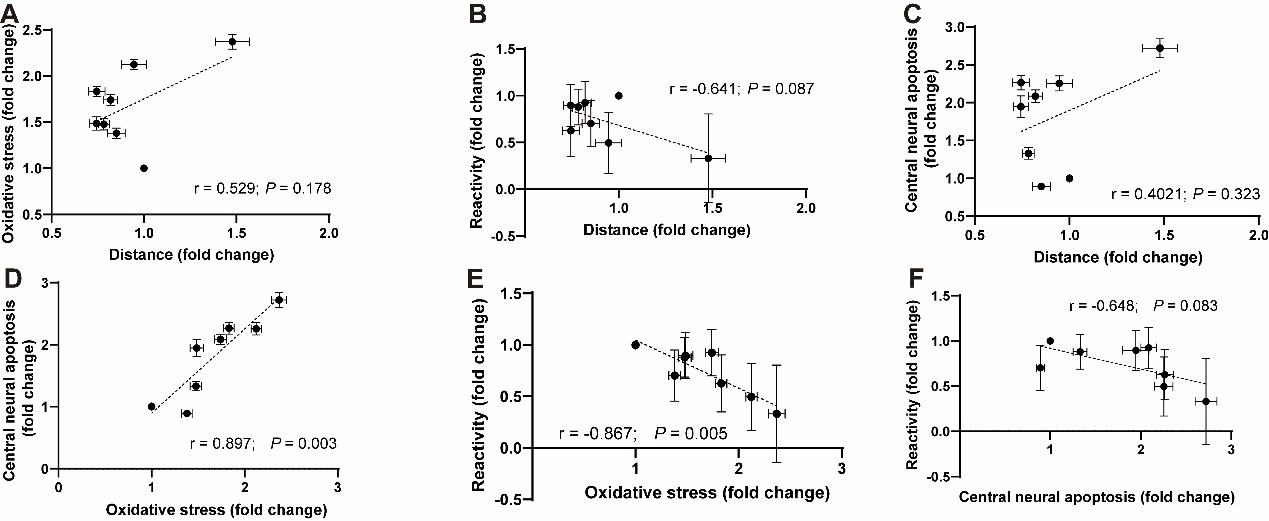


**Supplementary Fig. 2: Representative correlation plots supplement Fig. 5B.** Correlations between distance and oxidative stress (A), reactivity (B) and central neural apoptosis (C); D-E: Correlations between oxidative stress and central neural apoptosis (D) and reactivity (E); F: Correlation between central neural apoptosis and reactivity.

**Supplementary table 1: Instruments & consumables**

| **Product** | **Cat No** | **Producer** |
| --- | --- | --- |
| Dissecting microscope | SZX7 | OLYMPUS, Japan |
| CCD camera | VertA1 | Shanghai Tucson Vision Technology Co., Ltd., China |
| Precision electronic balances | CP214 | OHAUS, USA |
| 6-well & 96-well plate |  | Nest Biotech China |
| Behavior analyzer | V3.11 | ViewPoint Life Sciences, France |
| Multifunctional microplate reader |  | SPARK, TECAN, Switzerland |
| 1-(3-Chlorophenyl) piperazine hydrochloride | 52605-52-4 | Shanghai Aladdin Biochemical Technology Co., Ltd., China |
| Fish Cortisol ELISA KIT | CSB-E08487f | MBS, USA |
| Methylcellulose | 9004-67-5 | Shanghai Aladdin Bio-Chem Technology Co., Ltd., China |
| dimethyl sulfoxide (DMSO) | 67-68-5 | Sigma, Switzerland |
| Menaquinone | I1817117 | Shanghai Aladdin Biochemical Technology Co., Ltd., China |
| Specific ROS fluorescent staining kit | C10492 | CellROX^TM^ Green Reagent, Invitrogen, USA |
| Mycophenolate morph ethyl ester | 115007-34-6 | Shanghai Aladdin Bio-Chem Technology Co., Ltd., China |
| Acridine Orange | 494-38-2 | Shanghai Aladdin Bio-Chem Technology Co., Ltd., China |

**Supplementary table 2: Sample anxiety relief efficacy concentration maximum detection concentration (MTC). (n = 10)**

| **Group** | **Concentration （μg/mL）** | **Death（tail）** | **Mortality Rate（%）** | **Phenotype*** |
| --- | --- | --- | --- | --- |
| Control | - | 0 | 0 | NSA |
| Anxiety model | - | 0 | 0 | NSA |
| Stachyose | 125 | 0 | 0 | NSA |
|  | 250 | 0 | 0 | NSA |
|  | 500 | 0 | 0 | NSA |
|  | 1000 | 0 | 0 | NSA |
|  | 2000 | 3 | 10 | NSA |
| PS128 | 125 | 0 | 0 | NSA |
|  | 250 | 0 | 0 | NSA |
|  | 500 | 0 | 0 | NSA |
|  | 1000 | 0 | 0 | NSA |
|  | 2000 | 0 | 0 | NSA |
| WP | 31.2 | 0 | 0 | NSA |
|  | 62.5 | 0 | 0 | NSA |
|  | 125 | 0 | 0 | NSA |
|  | 250 | 0 | 0 | NSA |
|  | 500 | 0 | 0 | NSA |
| DC | 125 | 0 | 0 | NSA |
|  | 250 | 0 | 0 | NSA |
|  | 500 | 0 | 0 | NSA |
|  | 1000 | 0 | 0 | NSA |
|  | 2000 | 0 | 0 | NSA |
| WP + Th | 7.04 | 0 | 0 | NSA |
|  | 14.1 | 0 | 0 | NSA |
|  | 28.2 | 0 | 0 | NSA |
|  | 56.3 | 0 | 0 | NSA |
|  | 112.6 | 10 | 33 | NSA |

*: Non-significant abnormality

**Supplementary table 3. The summary of effects of nutrients and combinations**


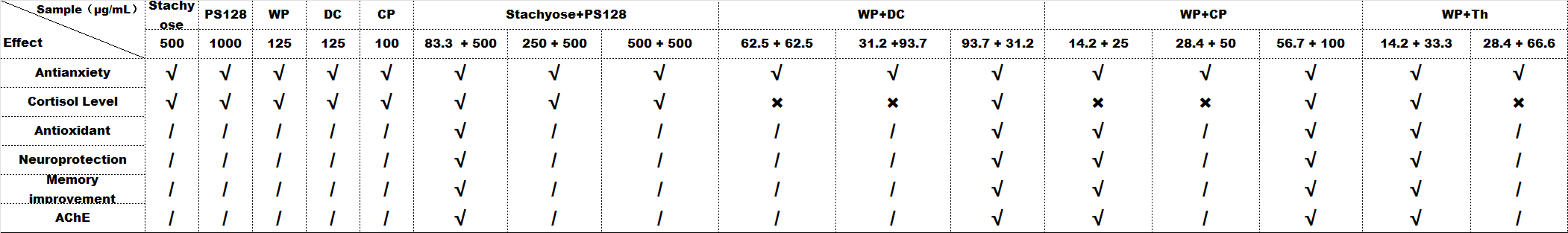


√：Effectivity

×：Invalidity

/：Not Applicable
